# A qualitative study of the aspirations and challenges of low-income mothers in feeding their preschool-aged children

**DOI:** 10.1186/1479-5868-9-132

**Published:** 2012-11-16

**Authors:** Allison N Herman, Khushi Malhotra, Gretchen Wright, Jennifer O Fisher, Robert C Whitaker

**Affiliations:** 1 Department of Public Health, Center for Obesity Research and Education, Temple University, 3223 North Broad Street, Suite 175, Philadelphia, PA, 19140, USA

**Keywords:** Feeding, Child, Preschool, Obesity, Parenting, Eating behavior, Qualitative evaluation

## Abstract

**Background:**

The prevalence of obesity among preschool-aged children has increased, especially among those in low-income households. Two promising behavioral targets for preventing obesity include limiting children’s portion sizes and their intake of foods high in solid fats and/or added sugars, but these approaches have not been studied in low-income preschoolers in the home setting. The purpose of this study was to understand the contextual factors that might influence how low-income mothers felt about addressing these behavioral targets and mothers’ aspirations in feeding their children.

**Methods:**

We recruited 32 English-speaking women in Philadelphia, Pennsylvania who were eligible for the Supplemental Nutrition Assistance Program and who were the biologic mothers of children 36 to 66 months of age. Each mother participated in 1 of 7 focus groups and completed a brief socio-demographic questionnaire. Focus group questions centered on eating occasions, foods and drinks consumed in the home, and portion sizes. Each focus group lasted 90 minutes and was digitally recorded and transcribed verbatim. Three authors independently identified key themes and supporting quotations. Themes were condensed and modified through discussion among all authors.

**Results:**

Thirty-one mothers identified themselves as black, 15 had a high school education or less, and 22 lived with another adult. Six themes emerged, with three about aspirations mothers held in feeding their children and three about challenges to achieving these aspirations. Mothers’ aspirations were to: 1) prevent hyperactivity and tooth decay by limiting children’s sugar intake, 2) use feeding to teach their children life lessons about limit setting and structure, and 3) be responsive to children during mealtimes to guide decisions about portions. Especially around setting limits with sweets and snacks, mothers faced the challenges of: 1) being nagged by children’s food requests, 2) being undermined by other adults in the family, and 3) having bad memories from childhood that made it hard to deny children’s food requests.

**Conclusions:**

Although the primary aspirations of low-income mothers in feeding their preschool-aged children were not focused on children’s weight, these aspirations were compatible with obesity prevention strategies to limit children’s portion sizes and their intake of solid fats and/or added sugars.

## Background

The prevalence of obesity among preschool-aged children is increasing [1,2], and this trend has stimulated efforts to prevent obesity early in life [3,4]. The few randomized trials of obesity prevention interventions in early childhood have shown limited success [5,6]. To prevent excess energy intake, two promising behavioral targets are to limit children’s portion sizes [7] and their intake of solid fats and/or added sugars, especially from foods that are relatively high in energy and low in nutrients [8,9].

Although there have been laboratory-based behavioral studies showing a reduction in young children’s energy intake by altering both the portion size and energy density of foods [10-12], there has been little translation of these strategies to community-based interventions [13]. Mothers of preschool-aged children are a logical target group for this translation effort because approximately 70% of the energy intake of preschool-aged children occurs in the home [14], and mothers are the adult caregivers with the primary responsibility for feeding children at this age. A further focus on low-income households is warranted because children in these households are at greater risk of obesity [15].

To develop interventions that help low-income mothers serve appropriate portion sizes to their preschool-aged children and reduce their children’s intake of solid fats and/or added sugars, more qualitative research is required to understand the household context in which the intervention would occur [16]. Previous qualitative research in this population has identified some important contextual factors related to child feeding. For many low-income mothers, obesity is of lower concern than their child’s social, emotional, or cognitive development, and mothers do not necessarily view feeding their child as related to these developmental domains [17]. Traditional approaches to delivering information about healthy feeding practices have failed to engage low-income mothers, who may feel they are receiving a lecture on how to parent [18]. These approaches can leave mothers feeling judged about their parenting and implicitly blamed for their child’s body weight [17,19-21].

Feeding preschool-aged children is an integral part of parenting and the mother-child relationship, and feeding often evokes conflicting emotions in mothers. For example, if children do not eat enough, mothers may feel unable to nurture their children, and if children eat too much, mothers may feel unable to protect their children against obesity [21]. Therefore, interventions that ask mothers to change how much (portion size) and what (foods high in solid fats and/or added sugars) children eat necessarily involve the emotionally sensitive subject of parenting and the mother-child relationship. Accordingly, appeals to emotion are required to change these practices. Evidence-based recommendations to mothers that sound logical are unlikely to alter feeding practices if the messages do not engender positive feelings in mothers about their relationships with children [22]. In addition, messages about feeding may seem insensitive and evoke negative emotional reactions if the messages are not responsive to the practical constraints and competing demands of mothers’ daily lives [19].

To develop an obesity prevention intervention that would result in sustained changes in how much and what mothers feed their preschool-aged children, we sought to identify parenting aspirations that were positive and emotionally compelling and that could be linked to feeding practices. To accomplish this, we undertook a qualitative study using focus groups. Rather than approach the broad topic of parenting, which could identify aspirations unrelated to feeding, we asked mothers open-ended questions about their feeding practices. We explored mothers’ rationale for these practices and their emotional responses to alternative practices mentioned by other focus group participants or the group leader. This approach was intended to provide an understanding of: 1) what mothers did (when, what, and how much) in feeding their children, 2) why mothers did what they did, and 3) mothers’ emotions about feeding. This report focuses on mothers’ aspirations that influenced feeding practices and the challenges faced by mothers in achieving those aspirations.

## Method

### Setting and investigators

We conducted focus groups with mothers of preschool-aged children living in low-income households in Philadelphia, PA. The mothers were recruited from clinics of the Special Supplemental Nutrition Program for Women, Infants, and Children (WIC). WIC is a federal program, administered by the U.S. Department of Agriculture (USDA), that provides supplemental food, nutrition counseling, and referrals to low-income women and to infants and children up to 60 months of age, who are found to be at nutritional risk [23]. WIC clinics were used to locate mothers with preschool-aged children in families receiving benefits from the Supplemental Nutrition Assistance Program (SNAP), formerly the Food Stamp Program, another USDA-administered program that provides food assistance to families with incomes at or below 130% of the federal poverty line [24].

The five authors formed an inter-disciplinary research team: two senior investigators (RW and JF) combining backgrounds in public health, pediatrics, nutrition, and human development; the research coordinator (GW), whose background was in sociology and child development; and two graduate students (AH, KM) in the fields of public health and social work. The focus groups were led by one of us (GW), who had 10 years of experience in qualitative data collection involving low-income families. The study design and data analysis were led by a senior investigator (RW) who had over 15 years of experience conducting qualitative research with low-income mothers.

### Participant recruitment

To recruit participants, a member of the research team approached women in the waiting rooms of WIC clinics. The research team recruited participants during hours with a high volume of visits and all women in the waiting rooms were approached. The women were provided information about participating in a study on how mothers make decisions when feeding their preschool-aged children. Flyers were also posted in the WIC clinics and contained study information and a contact phone number. Of the 88 women who expressed interest in participating (12 by phone), 70 met the eligibility criteria for the study. Each participant was required to: be the biologic mother of a 36- to 66-month-old child without a chronic medical condition affecting growth or eating (e.g., food allergies or intolerances, developmental disorders, or birth defects); receive or be income-eligible to receive SNAP benefits; be 18 years of age or older; have primary responsibility for feeding the preschool-aged child; and, be English speaking. All 70 eligible mothers were invited to a focus group, and 38 were able to attend one of eight scheduled groups, the first of which was a pilot group involving 6 participants. We chose to conduct focus groups instead of one-on-one interviews because focus groups allowed mothers to respond to the perceptions of other mothers, rather than only to the questions posed by the facilitator, and data could also be collected more efficiently. The data from the pilot group were not included in our analysis. The participants provided informed consent and were compensated with $40 for their participation. The Institutional Review Board of Temple University approved this study.

### Data collection

Seven focus groups with a total of 32 mothers were held at Temple University between October and December 2011. To minimize social desirability bias from the participants, the focus groups were held at Temple University rather than at the WIC clinics from which participants received nutrition counseling and food assistance. In addition, the facilitator opened each group by emphasizing, “You are the expert on your child, not us”. The groups ranged in size from 2 to 8 participants, with an average of 4 or 5 participants per group. The interview guide (Table 1) focused on three domains: 1) eating occasions, with an emphasis on distinctions between meals and snacks; 2) foods and beverages in the home, with an emphasis on those high in solid fats and/or added sugars [8,25]; and 3) portion sizes, with an emphasis on how mothers determined children’s portion sizes. The focus groups were conducted in a large conference room that allowed all the authors to observe unobtrusively while sitting apart from the table at which the participants and facilitator had their dialogue. The facilitator’s extensive experience in qualitative data collection and the small number of participants for each group allowed each participant ample opportunity to participate in the group discussion. Each group was digitally recorded and lasted 60 to 90 minutes.

**Table 1:** Sample questions from the focus group guide

**Domain**	**Sample questions**
**Eating occasions**	When I say the word “snack,” what do you think of? What does that word mean to you? How is that different than a meal?
	When I say the word “sweets,” what do you think of? What does that word mean to you? How about the word “dessert?”
	When you are with your child, how much control do you feel like you have over what and how much your child eats? When you are not with your child, how much control do you feel like you have?
	When do you give your child a snack? Who decides when your child has a snack? If you decide, how do you decide?
**Foods and beverages in the home**	Name some drinks that your child really likes and drinks often. What kind of milk does your child usually drink? Whole milk, 2%, 1%, skim?
	Name for me your child’s favorite things to have for a snack.
	Is there a favorite snack [sweet/ dessert/ drink] that you think your child should eat less often? What happens if you try to limit or cut down?
	What would happen if you try to change the type of milk your child drinks? Let’s say from 2% to skim?
**Portion sizes**	Who decides how much food is put on your child’s plate? Does your child have any say about this? Let’s take something like a pasta dish or spaghetti, as an example. How do you decide how much to give your child? Who serves the portion? You or your child? Do you think preschool-aged children should be allowed to serve themselves?
	What do you do if your child refuses to have certain foods even put on his or her plate, like a vegetable you have prepared? What do you do if a child wants more food than you put on his or her plate?
	Who decides how much of a drink your child has? If you decide, how do you decide? Let’s use juice as an example. If you are pouring juice from a larger container, who does the pouring? How do you decide how much juice your child gets to drink?
	Who decides how much your child eats for a snack [or how much of a sweet/dessert your child gets]? If you decide, how do you decide? If a snack comes in a package that is usually meant for one person, like a piece of candy or a bag of chips, how do you decide how much to give your child? What if your child wants more? Can your children get snack food without asking you?

After the focus group, each participant completed a brief survey that was administered by an interviewer in a private room. The survey consisted of demographic questions on maternal age, relationship status, race/ethnicity, education, household composition, and self-reported height and weight. It also contained the 6 questions from the short form of the US Household Food Security Survey Module [26]. No data were collected on the children’s weight.

### Data analysis

The focus group recordings were transcribed verbatim and the electronic transcript documents were imported into Atlas.ti (Scientific Software Development, GmbH, Berlin, Germany), version 6, a software program that assists in organizing and analyzing qualitative data. The data analysis was inductive and followed the constant comparative method [27]. Our goal was to identify common themes about the social, emotional, cultural, and economic context of households that appeared to influence the decisions made by mothers regarding children’s portion sizes and their intake of solid fats and/or added sugars. The identification of themes was informed by our knowledge of contextual influences identified in other qualitative studies (e.g., [17,20,28-31]) and by proposed frameworks about how the household context affects the relationship between parenting and feeding (e.g., [32-34]). We did not, however, employ a deductive approach to identify evidence to support a set of *apriori* themes or an existing theoretical framework about child feeding. After seven focus groups were held we achieved saturation in our targeted content areas. Comments made in some groups about family meals and how they influenced mothers’ relationships to their children warranted the development of a new discussion guide. We held four focus groups on this topic and those results will be published elsewhere.

Immediately after each focus group, all authors met to discuss their impressions of the issues raised by mothers in the focus group. However, we did not develop a list of themes meant to guide later coding of the transcripts. After all seven focus groups were completed, three of us (GW, KM, AH) independently read each transcript two times, identified a set of themes, and designated transcript text that supported each theme. The three transcript coders then met to achieve consensus about themes and condense them into a smaller group of themes. In this process, the group agreed on theme names, merged related themes into single themes, and dropped themes for which there was insufficient evidence across focus groups. The two of us who did not code transcripts (RW and JF) verified that the themes were supported by selected focus group quotations. We report here on a subset of related themes which describe mothers’ aspirations that influence their feeding practices and the challenges related to those aspirations.

From the survey data, we computed descriptive statistics using SPSS (IBM, Armonk, New York), version 19. Maternal self-reported weight (pre-pregnant weight if pregnant) and height were used to calculate body mass index (BMI; weight in kilograms divided by the height, in meters, squared). The food security scale was scored according to the USDA guidelines, and mothers with a score of 2 or greater (out of 6) were considered to be in food insecure households [26]. Because we could not link voices on the focus group transcript to individual participants, we did not link participant comments in the focus group to characteristics obtained from the questionnaire.

## Results

The mothers in this study were predominately black and almost half had no education beyond high school (Table 2). While over 80% (26) were unmarried, 69;% (22) were living in a household with another adult and 41% (13) with one or more of the child’s grandparents. Approximately one-fifth were food insecure and over half were obese (body mass index ≥30 kg/m^2^).

**Table 2:** **Characteristics of mothers in the focus groups** (**N** = **32**)

**Characteristic**	**Mean (Range)/N (%)**
Age, years	27.5 (20–41)
Race	
Black	29 (91)
Other, non-White	3 (9)
Latina/Hispanic ethnicity	
Yes	1 (3)
No	31 (97)
Education	
Less than high school	4 (12)
High school graduate or GED	11 (35)
Some college or technical school	15 (47)
College graduate	2 (6)
Currently married	6 (19)
Ever married^1^	7 (23)
Married to father of preschool-aged child	3 (9)
Living with father of preschool-aged child	11 (34)
Number of people in household	5.3 (3–11)
Number of adults in household	2.6 (1–8)
Number of children in household	2.7 (1–5)
Child’s grandmother lives in household	9 (28)
Child’s grandfather lives in household	5 (16)
Mother’s adult partner or husband lives in household	17 (53)
Other adult friend or relative lives in household	8 (25)
Food insecure	7 (22)
Body-mass-index^1^	
18.5–24.9 kg/m^2^	9 (29)
25.0–29.9 kg/m^2^	5 (16)
30.0–34.9 kg/m^2^	8 (26)
>35.0 kg/m^2^	9 (29)
Age of preschool-aged child, months	50.9 (36.9–65.9)
Sex of preschool-aged child	
Female	15 (47)
Male	17 (53)

GED = General Education Diploma.

1 Missing data for one participant.

We identified three themes about maternal aspirations that influenced feeding practices (Table 3) and three themes about challenges in achieving these aspirations (Table 4). The six themes are described below. We later discuss how they might inform the development of obesity prevention interventions for low-income preschool-aged children.

**Table 3:** Supporting quotations for themes related to aspirations that influence feeding practices

**Theme**	**Supporting quotations**
Preventing hyperactivity and tooth decay	So the first time she drank Pepsi, I literally thought my husband was drinking my sodas at night, and I was getting mad. But one time I caught her. She was like nine months and unscrewing the top to the Pepsi and taking it to the head. That’s what made me stop drinking Pepsi because I was wondering why she was always hyper late at night.
	He had two cavities. They told me they wanted to take his two front teeth out. So, I said no because, I actually have a niece who is four-years-old and they went to the doctor at the same time, and they told us that we have to take their two front teeth out. I said, “No way!” My niece is four-years-old and she got her two teeth out, her two front teeth. I don’t like that. My son still has his teeth. Because they said, “Ok, ma’am, I understand you don’t want his teeth out, so, we’re going to go another route.” So they capped them. So, I was not having that. Couldn’t have his teeth out.
	I’m not a big sugar kicker. I feel like the sugar keeps them going! If it’s 100% juice I feel like they’re getting the fruit out of it but, as far the sugar content, I don’t want to deal with that all day! So they get the flavor, but I still dilute it and she’s four.
Teaching life lessons to children	I buy a case of water and the packets of the juice. I count- you get a juice with your dinner and then you get another juice- that’s the only juice you’re going to get, two juices out of the day. The rest you have to drink water.
	So she got the cookie or the cake or the cupcake, but I feel like it gives me something, if I feel like she didn’t eat that fruit that I sent in the snack pack for lunch or she didn’t drink enough milk or something, it’s like a trade-off. You don’t get the cookie.
Being responsive to children	They won’t eat peas, for some reason. They say it looks nasty to them so they don’t eat peas. So I won’t cook peas for them, so I know they only like corn, broccoli, string beans and maybe greens.
	Sometimes I’ll give my kids two [juice boxes] in a row. If I see that they guzzled the juice down really fast, I can see they’re really thirsty, so I’ll say, “Go ahead, you can have another one”.
	Knowing your child is to understand, where they stand, and how they function and when you can kind of judge.

**Table 4:** Supporting quotations for themes related to challenges in achieving aspirations

**Theme**	**Supporting quotations**
Being nagged by children for sweets and snacks	So I shouldn’t have told him, “What do you want?” I always do that, “What do you want?” because if I get him something he doesn’t want, he throws it. Eventually I got up and got the chips for him that he wanted. And I gave it to him. Like the [social] worker was like, “You have to show him who’s the boss,” but my whole thing is it’s impossible with a 3 year old.
	It’s pretty much about snacks cause my son, he’s a con artist so he, you know, use “Oh, I’ve been in school”. He says, “Mom-mom or pop-pop, can you give me some money? I’ve been good.”So he tries to trick them but I’m right on top of him and try not to let him eat a lot of snacks.
	I give in sometimes. I give in to her because she looks like me. Her eyes are big and she just bats her eyes and she’s like, “Ma, please?” And then I’m like, “Okay”.
Being undermined by other adults in the family	When my kids are either at my mom’s or my sister’s, which is like, I guess, a grandparent syndrome, they get whatever they want and my in-laws, the same thing, we went down there, she gave him cake and ice cream for breakfast.
	My mom sneaks in and gives her juice, and she fills it up, and then I’ll be wondering like, “Why is he so hyped? I didn’t give him any juice.” Then my mom will look at me all crazy out of the side of her eye. She filled the cup up and gave him a big cup, too.
	I just try to change the sugar content if she’s going to drink a large amount of it. I have control over that, except when she’s around grandmom, they give her soda and coffee. Why are you offering a little girl coffee in the morning or before bed? Like, that’s not decaf. Coffee, this is like caffeinated, dark roast, sugar and cream.
Having bad memories from childhood makes it hard to say “no”	But being a mother of five is the best thing that pretty much ever happened to me in my life because of the way I was raised. My childhood was kind of rough and, you know, my children think that they’re being fed with a silver spoon. I want them to feel special because my children are really special to me. My children are very demanding and I try to give them what they want, for the most part.
	I just want my children to have the things that I didn’t have. I didn’t have the choice to ask or, you know, I mean I can’t speak for everyone in this room but my childhood wasn’t very good growing up. So I just try to give them the highlights and things that I didn’t have.

### Aspirations that influence feeding practices (Table 3)

#### Preventing hyperactivity and tooth decay

Mothers expressed a strong desire to limit children’s sugar intake because of their concern about sugar causing hyperactivity and tooth decay. One mother stated, “I can’t take the madness anymore with these kids and sugar”. Another mother recounted the following story:

"“One time [my son] went to my aunt’s house and came back and would not calm down. And I said, ‘What did they give him?’ I had to call her. I said, ‘He is entirely too hyped. What is going on with him? Why is he jittery? He can’t sit down? What is going on?’ She said, ‘Oh, yeah, we had some fruit snacks, and we had the regular fruit snacks, not the natural kind.’ I’m like, ‘Oh, Lord. So that’s what it is. All that sugar’”."

Mothers also described their children or relatives’ young children having cavities. In describing what foods were appropriate for her children’s aunt to bring into her home, one mother said, “She knows I don’t want my kids eating candy, because I don’t want their teeth all messed up”. Another mother described eliminating candy after her older son had his decayed teeth extracted.

"“My six-year-old, they used to spoil him. His dad used to give him all types of candy, juices and stuff, and he had to get his teeth pulled out at the age of three, his front teeth at the age of three. And my other two little ones, I was like no, cannot have that done, so that’s why I don’t give them candy. But that’s why the candy thing came out because my son had to get his teeth taken out at three because they started rotting from all the candy and juice and stuff”."

#### Teaching life lessons to children

By setting limits and saying “no” to foods that their children wanted, mothers hoped they could teach their children an important life lesson about not always being able to have what you want or have it when you want it. In speaking about saying “no” when her child wanted more food, a mother said, “Sometimes they get mad and fall out but, that’s something you have to deal with. We don’t always get what we want. That’s life. No.” Mothers acknowledged the emotions they experienced in saying “no,” but explained their rationale for doing so. One said:

"“Sometimes it hurts you as a parent more than the child when you say no. I don’t know why though. I don’t like my son looking all upset or crying, but I also explain to him and say no sometimes because if you don’t have it and the child expects it, if everything is always yes, then it’s going to be a problem when you don’t have it”."

Another mother spoke of how she changed her approach to her children’s requests for food to teach her children about working for things and earning them.

"“So my [first] thing was I wanted them to always have because I wasn’t able to have. But it kind of backfired, so now I’m trying to teach them that you have to earn what you’re getting. Whether it’s something small, you have to earn it by being good. I do say no a lot and I feel bad, but you can’t always just tell them yes, yes, yes, yes, yes, because they’re going to think they can always get what they want when they have to earn it. I know they’re three and four but you have to teach them this small, you know, so they’ll know when they get older”."

Another mother spoke of her long-term goal in saying “no” to her children’s request for food.

"“It’s not hard for me to say no because sometimes, you know, what’s good to you is not good for you. So I’m looking out for their wellbeing by saying no. So you might not like me right now or we might not get along for a little while, but you’ll love me later”."

In addition to imparting important life lessons by saying “no,” mothers also expressed the desire to provide some structure for children around eating by having rules about what and when they should eat. While it was not always clear how often mothers were able to uphold these rules, many mothers spoke of the importance of their children having some structure around eating. For example, mothers described rules about the kinds of foods children were expected to consume.

"“So I’ll give her that [peanut butter and jelly or chicken nuggets], but she has to eat a vegetable. When I’m home, you have to eat vegetables in my house. It’s always at a meal. Even if it’s not vegetables, at breakfast time, she has a fruit cup. It has to be something, it has to balance out”."

In addition to having some rules about what foods children should eat, mothers also described some rules they enforce regarding when children can eat.

"“Yeah, they have to ask me first and if I say ‘no,’ that means no, point blank. I don’t care if you’re mad at me, I don’t care if you cry. You can’t have it. At a certain time, like after 7:00, they can’t have any snacks”."

#### Being responsive to children

Mothers perceived that being responsive to their children’s mealtime eating patterns was a positive part of their relationship with their children. In speaking about mealtime portion sizes, mothers indicated that they knew how much food to serve their children at meals based on observing their children’s eating patterns. One mother said, “It seems like you know how much to put on their plate because you know how much they’re going to eat. As a mother, you already know how much to give them”. Mothers repeatedly indicated that children were unique with regard to their food preferences—how much of each type of food they would usually eat on any given day. Mothers appeared to honor and value this expression of their child’s individuality and wanted to be responsive to it. They often trusted their children’s ability to listen to their own bodily cues. One mother said, “My son knows when to stop [eating pizza]. He stops on his own”. Other mothers allowed their children to participate in determining portions sizes. One described her approach as follows, “I call them in the kitchen, and I ask them, ‘Do you think this is enough or is this too much?’ They may say yes or no, however. Then I give them their plate and they set it down.” While mothers were responsive to children around determining mealtime portions, mothers were also clear in their belief that adults should set limits with children around sweets and snacks. Yet, they experienced this as a major challenge.

### Challenges in achieving aspirations (Table 4)

#### Being nagged by children for sweets and snacks

Mothers struggled to say “no” to their children’s frequent requests for sweets or snack foods. Many mothers felt exasperated by their children’s nagging and frustrated with themselves for “giving in” to the nagging. One mother described her situation as follows:

"“It’s just annoying, they’ll just keep asking, especially my daughter. She keeps asking, keeps asking, keeps asking, ‘Can I have a snack? Can I have a snack? Can I have a snack?’ You just be like, ‘Just go ahead, just go ahead, just go ahead.’ Then I wonder, didn’t I just say no?”"

On the other hand, one mother tried to explain to the others in the group her own resolve about being firm with her children. She said, “I don’t think you always should have to prove a point to your kids. When you say no, it should be no…. If you mean no, the answer is no. You don’t have to bite your words just to say no”.

Mothers also described their children as being clever in the ways they managed to convince other adults to give them sweets and other junk food. For example, one mother of two preschool-aged boys noted the struggle with her children getting sweets from her own mother and her preschoolers’ father while she was not home.

"“I can fix something, put it on the stove, but when I come back, it’s still there. My kids are running around like they had a ton of sugar, because my mom and their father give the kids everything. The kids have this whine and they pout and my mom and their father can’t stand it and they are like, ‘Here. Just take it. Go.’ The kids are eating cookies and cake and I’m like, ‘Here’s the food, why didn’t you feed them?’ ‘Well, they wanted… .’ No, it’s not what they want. You have to give them the food I made”."

#### Being undermined by other adults in the family

Other adults in the home, instead of providing support to mothers around rules and structure in feeding young children, tended to undermine the mothers’ authority in this aspect of parenting. Over half of participating mothers reported living with an adult partner or their husband. However, mothers’ frustration at being undermined centered on other adult relatives in the home, especially, grandparents. One mother referred to this as the “grandparent syndrome,” and this expression resonated deeply and immediately with others in the focus group in which it was mentioned. The most challenging issue for mothers was that other adults offered children junk food throughout the day. A mother living with her own parents described her dilemma in the following way:

"“If I say no, it’s kind of hard with me because it’s like my dad and my son have like this really special bond. So if I say no my dad feels like, ‘Well, that’s my grandson.’ I can’t argue with my dad. So if I don’t give my son what he wants, he still has that other source”."

The challenges even involved three generations of mothers in one home. One mother explained that both her mother and her grandmother tended to spoil her son by feeding him sweets. She explained the situation as follows:

"“The worst ever is with my mom’s mom, because my mom does it but my grandmom feels like she did it with me so she’s going to do it with my son. And she’s at that whole stage where she’s like, ‘I’m not going to be here that much longer so I want them to love me and be happy with me’”."

Expressing her struggle with her own aunt living in the home, one mother noted:

"“I tried to [cut down on juice] but my aunt, she’s like, ‘Give it to her. I bought it for her.’ And then my daughter has a lot of cavities so I tried to cut down on it. But my aunt said, ‘They are going to fall out anyway, give it to her.’ So it doesn’t work”."

#### Having bad memories from childhood makes it hard to say “no”

A final challenge for mothers in achieving their aspirations was their own childhood memories surrounding adult authority. Mothers candidly described their own childhoods as “hard” or “rough.” They described often having few choices and almost always being told “no.” One mother said, “It’s just really hard when you have a bad childhood. You are very aware. Everybody told me no. This is my first child. You feel like, ‘Oh, well I didn’t have it so I want him to have it.’” Another mother stated her feelings succinctly in saying, “I wouldn’t want my mom telling me no, so I don’t want to tell them no about what they can eat. Well, the candy part, yeah, but other than that, [I don’t want to tell them] no.” This feeling was echoed by another mother who described her desire to give her children a different childhood than her own and why she felt guilty about saying “no”.

"“But sometimes I feel bad after I tell my kids no because I was used to me being told no, no, no, no when I was little. So I feel bad when I tell my kids, no, but it just all depends on what I’m telling them no to. I learned on my own what to say no to and what not to say no to because I was just being told no for everything I wanted. So when it comes to my kids, you know, I try to do things different with them than was done with me when I was growing up”."

## Discussion

### Summary of key findings

When we asked low-income mothers open-ended questions about feeding their preschool-aged children, mothers revealed aspirations which could help inform obesity prevention interventions. The mothers described a positive agenda for feeding that included having a calm child in good oral health by limiting children’s sugar consumption; teaching children life lessons by setting limits around junk food and providing structure to eating; and, being responsive to children’s mealtime eating patterns, which guided decisions about portion sizes.

These aspirations were unrelated to preventing obesity. We neither invited nor avoided the topic of obesity in our focus groups, but the discussion of obesity and the connection between feeding and obesity was notably absent. The lack of a discussion surrounding obesity in our focus groups is particularly notable, given that we began each focus group by saying:

"“We are interested in helping parents have their children grow up with a healthy weight. Why some children become heavy and others do not is still a bit of a mystery. People who have been trying to understand this think that a child can become overweight from eating too much food or the wrong kinds of food, but it is sometimes hard to know how much food and what kinds of foods children need to be healthy. We are interested in what you do and what you think when it comes to feeding your child”."

Mothers also described a household context that presented challenges in achieving this positive agenda, particularly in limiting sugar consumption. These challenges included being nagged by their children for sweets and snacks, being undermined by other adults in the family who gave children sweets and snacks, and having bad childhood memories that made mothers feel guilty about saying “no” to their children. Below we discuss our interpretation of these findings, place the findings in the context of prior research, note the limitations of our study, and suggest implications for designing obesity prevention interventions.

### Aspirations that influence feeding practices

Mothers had aspirations that were consistent with some behavioral goals in childhood obesity prevention—reducing children’s sugar intake, setting limits and structure around eating, and responsive feeding during mealtimes. Mothers wanted to limit their children’s sugar intake to avoid hyperactivity and tooth decay, which could be framed as an aspiration to have a calm child with a beautiful smile. There is some evidence linking sugar intake with dental carries [35], but there is little evidence linking it with hyperactivity [36]. Other qualitative studies showed that low-income mothers thought about a healthy child in ways that they did not directly relate to nutrition, such as a child with healthy skin and hair or one who was happy, intelligent, and well-behaved [21,28,37].

Mothers were also motivated to teach children life lessons by setting limits and providing structure around children’s eating. White and colleagues, in their focus groups with mothers of preschool-aged children across eight US states, noted that mothers valued teaching their children “lessons they’ll use for life” [31] (p.22). In contrast to our study, however, these lessons were about mothers and children cooking and eating together rather than the lessons our mothers noted about children learning to deal with limit setting and structure in eating.

In response to describing how they determined children’s mealtime portion sizes, mothers indicated that they were responsive to their children’s food preferences and took pride in intimately knowing and responding to those preferences. Mothers in other qualitative studies also suggested that they were guided in their portion size determinations by knowing their child and applying that knowledge to the particulars of an eating occasion, such as the food being eaten, the other foods being served, or the other foods eaten that day [20,37,38]. However, the evidence from qualitative studies is mixed about whether low-income mothers trust that their preschool-aged children know when they are full [31,37].

The question of whether mothers should trust children to decide how much to eat remains controversial [39-41]. A resolution of this controversy is suggested by the mothers in our study who, in general, expressed confidence in their children’s ability to know how much to eat as long as it was not sweets or other palatable snack food. Mothers nearly always served children their portions at mealtimes. However, in determining mealtime portion sizes, some mothers involved children in deciding how large portions should be, while others relied on their knowledge of how much food their children usually ate. In this regard the mothers were responsive to their children in determining portion sizes [42], but we have no information on whether these were age-appropriate portions. It was not a theme in our study, as in some others, that mothers prepared special or alternate meals to suit children’s food preferences [20,29,31].

We suspect that a latent aspiration reflected in mothers’ answers to questions about their feeding practices is the desire to build positive relationships with their children. In other qualitative studies of feeding practices in low-income mothers, the idea of building a positive relationship with children through feeding is often reflected in mothers trying to make children happy through food, regardless of whether this conflicted with the rules and limitations mothers wanted to establish [29,30]. Instead, our findings indicated that mothers wanted to build positive relationships with their children around food by being responsive to children while still establishing limits and structure, which are aspirations consistent with obesity prevention.

There is an emerging literature on maternal feeding styles and childhood obesity [43,44] that is based on a typology of four general parenting styles that arise from two dimensions of parenting, responsiveness and demandingness [32]. It was not our purpose to characterize feeding styles using this typology, and feeding was not observed. However, mothers in our study might be best characterized as aspiring to an authoritative feeding style in that they were generally responsive around mealtime portions and wanted to set limits (making demands). The fact that mothers were not always able to set limits due to contextual challenges supports the lack of consistency seen between reported and observed feeding styles [45]. In general, mothers in our study were invested in feeding. They did not appear to have a feeding style that was neglectful, uninvolved, or disengaged [46], but such a style would be less likely in those who volunteered for a study on perceptions about feeding their children.

### Challenges in achieving aspirations

A prior study showed that WIC health professionals felt mothers often had difficulty setting limits with their children in the feeding relationship [19], and this was also noted by low-income mothers in another study [29]. Our study reveals why low-income mothers say they aspire to set limits and the contextual factors that make limit setting hard to do. We and others have previously noted the influence of other adult family members in the mother-child feeding relationship, especially the mothers of young mothers [21,30,37,47,48]. In this study, we found that when mothers’ agenda to limit sweets and snacks was undermined by other adult family members in the home, mothers felt guilty about interfering and saying “no” because they did not want to harm the relationship between their child and the other adult. Sweets and snacks were a source of conflict in a three-way relationship between child, mother, and another adult family member in the home. Mothers felt frustrated by the way their preschool-aged children cleverly manipulated or nagged them and other adults to obtain sweets and snacks.

Mothers shared painful childhood memories about being told “no”. In one study that asked low-income mothers about their childhood experiences, mothers described their own parents as being either too strict or neglectful in their feeding practices [29]. In another study, low-income, Latina mothers connected the idea of gratifying children with food and good parenting [30]. Similarly, our mothers implied that in saying “no” they were failing to show love because this is how they experienced hearing “no” as a child. Mothers were then left with internal conflict about if and how to say “no” without making the child feel unloved. This could explain why mothers may not effectively set limits around feeding even though they aspire to do so.

### Study limitations

The convenience sample of 32 mothers who participated in the study was not necessarily representative of the low-income mothers in Philadelphia, or in other US cities, who met our inclusion criteria. All but one of the mothers, for example, identified themselves as black or African American. Mothers who volunteered to participate may also have devoted more time and energy to feeding their children than other low-income mothers.

Although there were not any direct references to food insecurity in our focus groups, mothers’ painful childhood memories of hearing “no” and the difficulty of saying “no” to their children may have stemmed from mothers’ material deprivation, including food insecurity, during childhood. Mothers may not have felt comfortable disclosing food insecurity during the focus groups, and we did not specifically ask probing questions about it during the groups. Other qualitative studies have suggested that present or past food insecurity may affect how mothers feed their children [30,49]. Aside from food insecurity, our study may have missed other social or economic challenges faced by low-income mothers in feeding their children. For example, because the focus groups were held in the daytime during the work week, mothers with full-time jobs or school responsibilities during those hours were under-represented, and we may have missed themes related to how time pressures influence feeding children.

We did not collect weight or height data on children, but the distribution of BMI among the mothers was comparable to that of US black women this age [50]. Our study was not designed to compare differences among mothers’ perceptions about feeding according to their weight or the weight of their children. A larger study with an alternative design would be required to identify such differences. The validity of our findings is supported by achieving data saturation across seven focus groups, using three independent coders, and achieving consensus about themes among five authors with varying disciplinary perspectives. Nonetheless, the perceptions and behaviors mothers reported do not allow us to make inferences about their actual behaviors at home [45].

### Implications for obesity prevention interventions

The healthy feeding practices mothers discussed—setting limits, providing structure, and being responsive—were motivated by parenting aspirations other than obesity prevention. Obesity prevention interventions that target the household feeding of low-income preschool-aged children might benefit from focusing on these aspirations. The mothers did not discuss these aspirations in the context of weight or obesity.

Facilitated discussion might be a mechanism to allow mothers to discuss their aspirations in feeding and to help each other identify and address the contextual challenges they face in achieving these aspirations. Research in the Massachusetts WIC program suggests that mothers enjoyed sharing and learning from each other through facilitated group discussion [22]. Video, which has been used successfully in other parenting interventions [51,52], may serve as a catalyst for facilitated discussion and help standardize such interventions.

Evoking and affirming mothers’ aspirations and the positive emotions that accompany them may lead to more sustained changes in household behaviors because mothers are likely to be most motivated by those goals and related values that matter to them [53,54]. If mothers identify their own agendas in feeding that are consistent with obesity prevention, though not necessarily motivated by it, mothers may be more able to overcome challenges in achieving those agendas.

Limit setting around sweets and snacks was a central challenge noted by mothers in achieving their feeding aspirations. Nagging children, unsupportive adults, and painful childhood memories all made it hard for mothers to say “no.” To address these challenges it may be helpful to draw on some of the positive aspirations of mothers. Interventions might try to empower mothers to set their agendas for feeding and to think of them as household policies and routines. These policies, when developed to achieve mothers’ own healthy agendas for raising children rather than someone else’s agenda, can help prevent children from nagging for sweets or snacks or being undermined by other adults. It can also help mothers enforce their policies and deal with the uncomfortable feelings that may occur when there is resistance to these policies from children or other household adults.

Mothers’ aspirations could be the basis for presenting suggestions about how to change children’s snacking patterns. For example, even if mothers do not or cannot keep sweets or snacks out of the home, mothers can plan snacks, much in the way they already plan meals, so that the time, place, and type of snack offered is proactively established by the mother and the snack is not given as a reaction to the child’s request. A routine for snacks might attract mothers because it fits within the context of teaching a life lesson about the need for structure. Mothers may also have to say “no” less often because the children expect, accept, and respect the structure. If mothers reduce nagging because they have proactively imposed a routine around snack time, then they might be more able to think of saying “no” as loving and teaching their children rather than depriving or neglecting them. In addition, if mothers plan snacks, they can more easily offer children a controlled choice—a choice between options that are all acceptable to the mother. In this way, mothers still maintain their power and authority but share it with children by offering a choice. Mothers have the opportunity to feel gratified because they are attuned and responsive to their children rather than feeling frustrated or guilty because they have given in to a nagging request for junk food. Planning snacks as part of the daily routine and using controlled choice would extend the aspirations of teaching structure and being responsive in feeding from mealtimes to snack time, where it appears that children had more power than mothers wanted them to have.

There are several controlled evaluations of obesity prevention interventions that involve some focus on diet and target preschool-aged children and their parents [5,6]. Although some of these studies focus on specific parent feeding strategies, only one [55] places a heavy emphasis on topics in general parenting (e.g., general parenting styles, building bonds with children, and establishing routines). However, none of these studies attempt to identify and focus on mothers’ larger agendas and aspirations in parenting and linking them to relevant obesity prevention strategies, like planning snacks.

The mothers in our focus groups appeared more able to impose structure and practice responsive feeding during mealtimes than during snack times. Although it was not a major theme, it was spontaneously suggested by some mothers that mealtimes were also an opportunity to bond with children and establish positive relationships with them. Because there is some evidence that eating family meals may protect against obesity in preschool-aged children [56], future research should attempt to further understand mothers’ aspirations about family meals.

It is not surprising that mothers in our study felt a tension about being responsive while also setting limits, because there is a controversy in the literature about how best to apply these approaches in feeding [39-41] and in overall parenting [57]. Much of the controversy appears to stem from how control is defined and when and how exerting control can be healthy or unhealthy for children’s development. As noted by Gronlick and Pomerantz, “Parents cannot allow children to go unrestricted, even while fostering their initiation and considering their input” [57] (p 166). Our research suggests that obesity prevention interventions that focus on limiting children’s portions and their intake of solid fats and/or added sugars must help parents use control in a way that is healthy and that allows children to become independent and capable eaters who can control their own food intake to maintain a healthy weight. To do this, mothers may need to realize that they have power over children’s eating and how to use that power along with kindness and warmth so that it is seen by both mothers and children as love. If mothers experienced the power of limit setting and structure in their own childhood without parental warmth, then this type of parenting could have felt mean or even abusive. This might explain why mothers felt uncomfortable settings limits with their own children and confused or angry when their own parents did not set limits or provide structure in feeding grandchildren. Interventions with mothers to address limit setting in their children’s eating might benefit from exploring how mothers experienced limit setting as children.

Control does not mean that mothers need to be harsh, intrusive, or domineering. Instead, control can be the application of structure to help children become capable [57]. Structure is characterized by expectations and rules that are understandable to the child and experienced as part of a routine. This type of control does not need to ignore children’s preferences. Applied to eating, this may mean helping mothers provide a structure about when and where children eat. Doing so gives children a sense of safety and predictability which they can experience as maternal love and wisdom. In the case of certain foods, such as snacks and sweets, it may also be necessary for mothers to place structure on what and how much children eat by constraining the food choices they provide and the amount of food served.

In conclusion, our qualitative inquiry with low-income mothers about feeding their preschool-aged children elicited aspirations about feeding that mothers did not necessarily link to their children’s weight, but which might motivate mothers to make sustained change in feeding behaviors that could, nonetheless, help prevent obesity in their children.

## Abbreviations

BMI: Body-mass-index; SNAP: Supplemental Nutrition Assistance Program; WIC: Special Supplemental Nutrition Program for Women, Infants, and Children; USDA: United States Department of Agriculture.

## Competing interests

The authors declare that they have no competing interests.

## Authors’ contributions

**AH** recruited participants; observed all focus groups; coded data; developed the final themes; analyzed the questionnaire data; wrote the initial draft of the manuscript; and coordinated revisions to the manuscript. **KM** recruited participants; observed the focus groups; coded data; and developed the final themes. **GW** helped develop the interview guide, the questionnaire, and the recruitment plan; recruited participants; facilitated the focus groups; managed the focus group data; coded data; and developed the final themes. **JF** obtained funding for the study; assisted with the design and development of the interview guide, recruitment screener, and questionnaire; and observed the focus groups. **RW** designed the study and supervised the subject recruitment, data collection, data analysis and writing of the manuscript; observed the focus groups; developed the final themes; and helped obtained funding. All authors have read, approved, and provided critical revisions to the final manuscript.
